# Resistance to venetoclax and hypomethylating agents in acute myeloid leukemia

**DOI:** 10.20517/cdr.2020.95

**Published:** 2021-03-19

**Authors:** Antoine N. Saliba, August J. John, Scott H. Kaufmann

**Affiliations:** 1 Division of Hematology, Department of Medicine, Mayo Clinic, Rochester, MN 55905, USA; 2 Department of Molecular Pharmacology and Experimental Therapeutics, Mayo Clinic, Rochester, MN 55905, USA; 3 Division of Oncology Research, Department of Oncology, Mayo Clinic, Rochester, MN 55905, USA

**Keywords:** Venetoclax, hypomethylating agents, resistance, acute myeloid leukemia, azacitidine, decitabine

## Abstract

Despite the success of the combination of venetoclax with the hypomethylating agents (HMA) decitabine or azacitidine in inducing remission in older, previously untreated patients with acute myeloid leukemia (AML), resistance - primary or secondary - still constitutes a significant roadblock in the quest to prolong the duration of response. Here we review the proposed and proven mechanisms of resistance to venetoclax monotherapy, HMA monotherapy, and the doublet of venetoclax and HMA for the treatment of AML. We approach the mechanisms of resistance to HMAs and venetoclax in the light of the agents’ mechanisms of action. We briefly describe potential therapeutic strategies to circumvent resistance to this promising combination, including alternative scheduling or the addition of other agents to the HMA and venetoclax backbone. Understanding the mechanisms of action and evolving resistance in AML remains a priority in order to maximize the benefit from novel drugs and combinations, identify new therapeutic targets, define potential prognostic markers, and avoid treatment failure.

## INTRODUCTION

Acute myeloid leukemia (AML) is a heterogeneous disease with diverse molecular profiles, clinical outcomes, and disease-specific mortality. In 2020, there will be an estimated 20,000 new cases of AML and 11,200 deaths due to the disease in the U.S.^[1]^. For decades, the cornerstone of treatment for primary AML has been induction therapy with cytarabine and an anthracycline followed by consolidation with high-dose cytarabine chemotherapy or hematopoietic stem cell transplant (HSCT). Most patients with AML respond to initial induction chemotherapy and achieve a complete remission (CR), with rates ranging between 40 and 90% in different studies^[2–5]^. However, long-term survival rates remain significantly lower at about 40%−50% for adults younger than 60 years of age and less than 10% in adults age 60 years and older^[5,6]^. The high rate of relapse in older patients has been attributed to several factors, including lower tolerance to chemotherapy, poor performance status, comorbid diseases, a higher incidence of secondary AML, a higher incidence of adverse karyotypes, and acquired drug resistance^[7,8]^. In addition, 20% of patients treated with standard chemotherapy experience primary induction failure^[9,10]^. Therefore, mechanisms of *de novo* and acquired resistance to chemotherapy remain central barriers to achieving long-term remission.

The biological basis for this therapy resistance has been extensively studied. Certain cytogenetic abnormalities and genetic mutations are predictive of clinical course, prognosis, and response to conventional chemotherapy or more novel targeted therapies^[11–14]^. For instance, the presence of the genetic abnormalities t(8;21) or inv(16)/t(16;16) is associated with a high rate of response to conventional combination chemotherapy and a favorable prognosis^[3,11]^. Conversely, a complex karyotype or aneuploidy is associated with resistance to chemotherapy and a poor prognosis^[6,11]^. Although the relatively high proportion of patients with cytogenetically normal AML limits the prognostic and clinical utility of these cytogenetically defined groups^[11]^, recent advances in the understanding of the molecular landscape of AML have simultaneously expanded our prognostic ability and facilitated the development of agents targeting specific actionable alterations, including mutations of fms-like tyrosine kinase 3 (FLT3) and isocitrate dehydrogenase 1 and 2 (IDH1 and IDH2)^[15–17]^. Agents that target these abnormalities, including IDH inhibitors (e.g., ivosidenib and enasidenib), FLT3 inhibitors (e.g., midostaurin, gilteritinib, quizartinib, and sorafenib), and others, have offered an opportunity for remission with AML harboring targetable mutations^[17,18]^. Because targetable genetic mutations are found in only a minority of patients with AML, the parallel introduction of apoptosis-inducing therapies has shown promise in overcoming conventional chemotherapy resistance and can be used irrespective of the genetic signature^[19,20]^.

The “hypomethylating agents” (HMAs) azacitidine (5-azacitidine) and decitabine (5-aza-2′-deoxycytidine) are nucleoside derivatives that are incorporated into DNA, where they inhibit DNA methyltransferases (DNMT). HMAs have been primarily used to treat AML patients who are 60 years of age and older or those who are unable to withstand the rigors of conventional chemotherapy. In comparison to conventional combination chemotherapy, single-agent HMA or low-dose cytarabine (LDAC) regimens are usually better tolerated and are associated with lower rates of treatment-related mortality^[21]^. However, response rates to HMAs alone are low (10%−50%), with a median survival of about 6–10 months^[21–25]^. The observation that antiapoptotic proteins, including BCL2, BCLX_L_, and MCL1, are frequently overexpressed in AML and are associated with resistance to chemotherapy, eventually led to the addition of venetoclax, an oral selective small molecule inhibitor of BCL2, to LDAC or HMA therapy, initially in older adults with primary or secondary AML who were ineligible for conventional chemotherapy^[26]^. Rates of CR or CR with incomplete hematologic recovery (CRi) were 54% for the LDAC/venetoclax regimen and 67% for the HMA/venetoclax doublet, with a significant extension in overall survival (OS) to a median of 10–18 months^[27]^. These findings were more recently confirmed by the results of the phase III randomized placebo-controlled trial showing superiority of the combination of azacitidine and venetoclax over single-agent azacitidine in a group of previously untreated patients with AML who were not eligible for standard induction chemotherapy^[28]^. With a CR/CRi rate of 66% in the azacitidine/venetoclax group *vs*. 28% in the control group, a significant prolongation in median OS was achieved (14.7 months *vs*. 9.6 months respectively)^[28]^.

Despite these promising results, about half of the responders to azacitidine/venetoclax relapse after a median duration of about 18 months^[28]^. Therefore, although the last few years have witnessed important advances in the treatment of AML, drug resistance continues to be an important clinical problem. As the armamentarium of therapeutic options continues to expand with agents, like the Nedd8 activating enzyme (NAE) inhibitors (e.g., pevonedistat) and the mouse double minute 2 homolog protein (MDM2) inhibitor KRT-232, being added to the HMA/venetoclax backbone, understanding the mechanisms of action and evolving resistance remains crucial in order to maximize the benefit from emerging drugs and combinations, identify new potential therapeutic targets, and determine potential prognostic markers. Hence, we review the individual actions of the HMAs and venetoclax as well as their individual mechanisms of resistance, and we then focus on potential mechanisms of resistance to the HMA/venetoclax combination.

## HYPOMETHYLATING AGENTS

### Mechanisms of action

The HMAs azacitidine and decitabine constitute the backbone of treatment of high-risk myelodysplastic syndromes (MDS) and chronic myelomonocytic leukemia (CMML)^[29–32]^. Whether used as single agents or, more recently, in combination with venetoclax, HMAs have shown clinical activity in patients with primary, secondary, and relapsed/refractory AML^[19,28,33]^.

**Figure 1.**
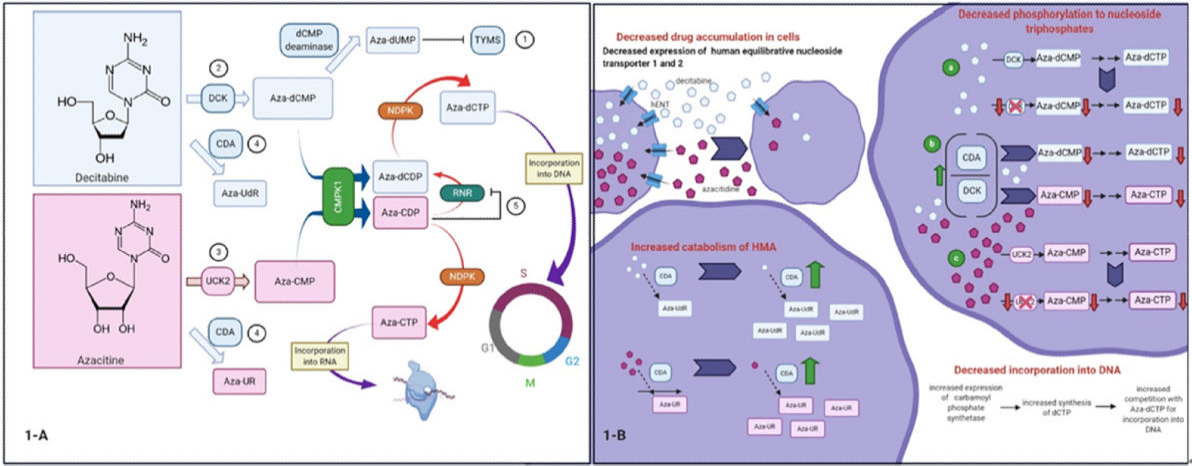
Proposed mechanisms of auto-resistance to hypomethylating agents^[35]^. A: mechanisms related to changes in nucleoside metabolism: (1) decitabine inhibits thymidylate synthase (TYMS). As a consequence dTTP levels decrease and dCTP levels increase. This is associated with a (2) decrease in deoxycytidine kinase (DCK), (3) increase in uridine/cytidine kinase 2 (UCK2), and (4) increase in cytidine deaminase (CDA). Alternatively, azacitidine is metabolized to aza-CDP, which (5) inhibits ribonucleotide reductase (RRM1). Inhibition of ribonucleotide reductase diminishes the conversion of aza-CDP to aza-dCDP and eventually aza-dCTP, which is capable of depleting DNMT1. In addition, dCTP levels decrease and, subsequently, (2, 4) DCK and CDA increase while (3) UCK2 decreases, which in turn decreases the conversion of azacitidine to aza-CMP; B: additional mechanisms of resistance. Decreased expression of human equilibrative nucleoside transporter (hENT) 1 and 2 is associated with decreased intracellular accumulation of decitabine and azacitidine. Although not a universal finding, a possible mechanism of resistance in MDS is an increase in carbamoyl-phosphate synthetase (CAD) expression, resulting in increased synthesis of dCTP, which competes with aza-dCTP for incorporation into DNA. CMPK: cytosine nucleoside monophosphate kinase; RNR: ribonucleotide reductase; NDPK: nucleoside diphosphate kinase. Figure was created with BioRender

HMAs are pyrimidine analogs of the nucleoside cytidine that showed promising cytostatic activity at higher doses in the 1960s^[34]^. In both azacitidine and decitabine, a nitrogen atom replaces a carbon atom in position 5 of the pyrimidine ring [Figure 1], but the sugar moiety is deoxyribose in decitabine and ribose in azacitidine^[35]^. There is evidence to support a dual mechanism of action for these agents: (1) a direct cytotoxic effect at higher doses, where the formation of covalent DNMT-DNA adducts leads to DNA damage; and (2) DNA hypomethylation and epigenetic modulation at lower doses with subsequent cell differentiation and tumor suppression^[36,37]^.

One of the ways HMAs exhibit their action is through epigenetic mechanisms^[38]^. The role of DNA methylation, specifically cytosine methylation, in the control of gene expression and epigenetic regulation was identified in different eukaryotic cells in the 1970s^[39–41]^. It was consequently thought that compounds capable of changing the patterns of cytosine methylation might impact cell differentiation and differential gene expression in various cell types that harbor the same genetic information. Azacitidine and decitabine were subsequently shown to alter differentiation in cultured mouse embryo cells^[42–44]^. Therefore, the focus with the use of HMAs shifted from their cytostatic and cytotoxic effects seen at higher doses to their role as potent inhibitors of DNA methylation at lower doses and with longer exposure^[45]^. Importantly, at these low doses, HMAs reportedly do not cause cell cycle arrest^[46]^.

Azacitidine and decitabine are prodrugs that require phosphorylation to monophosphate forms by uridine-cytidine kinase (UCK) and deoxycytidine kinase (DCK), respectively^[38,47]^. They are then further phosphorylated to diphosphate and triphosphate derivatives (5-aza-CTP and 5-aza-dCTP) by pyrimidine monophosphate and diphosphate kinases. These cytosine derivatives, like their natural counterparts, are also subject to degradation by cytidine deaminase (CDA).

The fates of 5-aza-CTP and 5-aza-dCTP are somewhat different. Approximately 80%−90% of azacitidine incorporates into RNA as 5-aza-CTP, resulting in alteration of RNA methylation and inhibition of protein synthesis^[48]^. The RNA-dependent effects of azacitidine are independent of the cell cycle and affect both messenger and transfer RNA^[49–51]^. The remaining 10%−20% of azacitidine is converted to 5-aza-dCTP by ribonucleotide reductase and, like the 5-aza-dCTP that is the main metabolite of decitabine, is incorporated into DNA.

Once 5-aza-cytosine (5-aza-C) is incorporated into DNA, the downstream effects are the same whether the altered base is derived from azacitidine or decitabine. When DNMTs act on normal cytosine, they form a transient covalent bond between a cysteine at the enzyme active site and C6 of cytosine. When these same enzymes act on 5-aza-C, the same covalent adduct is formed, but it is more stable and cannot be enzymatically reversed. As a result, cells are left without the enzymatic activity of the adducted DNMT enzyme molecule and with a new covalent DNA-protein crosslink for each DNMT molecule inhibited^[52–59]^. Thus, after HMA treatment, formation of DNA-protein cross-links and loss of DNMT activity are inextricably linked.

According to current understanding, HMA-induced hypomethylation of promoters of tumor suppressor genes leads to the interruption of feedback loops between DNA methylation and histone methylation. This reverses the silenced chromatin state of the histones at the tumor suppressor genes, thus facilitating their expression, the resulting suppression of leukemogenesis, and the induction of cellular differentiation^[45,60,61]^.

HMAs also trigger DNA damage response pathways. At high HMA concentrations, phosphorylation of histone H2A.x is observed^[62]^, indicating that one or more of the DNA damage-activated kinases ATM, ATR or DNA-PK have been activated. The irreversibly bound DNMT can also lead to cell cycle arrest at the transition from S to G2 through the activation of the ATM and ATR pathways^[63]^. The events that occur downstream of cell cycle arrest and lead to removal of the DNA-DNMT protein cross-links are still being elucidated^[64]^. Current understanding, enhanced by the recent demonstration of DNMT inhibitor/PARP inhibitor synergy^[62]^, suggests that DNMT-DNA covalent complexes might be removed by base excision repair, although the glycolyase involved and how it is activated remain to be determined. Inhibition of DNMT1 may also indirectly affect DNA repair mechanisms^[65,66]^. Vispé *et al*.^[67]^ showed that combining treatment with decitabine and DNMT1 siRNA uncouples DNA damage from DNA demethylation and again suggests a dual mechanism of action for HMAs.

### Activity of hypomethylating agents in myelodysplastic syndrome and AML

HMAs have been used for the treatment of myeloid malignancies for more than a decade. The role of HMAs is currently expanding across wider age groups for various therapeutic indications (including preemptive and maintenance therapies), in novel formulations (including oral), and in combination with multiple other agents (including venetoclax, lenalidomide, and IDH inhibitors)^[15,33,68–72]^. The initial observation that a variety of tumor suppressor genes are silenced as a consequence of promoter hypermethylation in MDS and AML prompted the investigation of HMAs as epigenetic drugs in these hematological diseases^[73]^. When initially tested in phase I studies in AML at higher doses (1500–2500 mg/m^2^ of decitabine per course), HMAs caused prolonged and dose-limiting myelosuppression with overall response rates of 30%−60%^[74,75]^. The mechanism of action at those higher doses was thought to reflect formation of DNA-protein cross-links, activation of the DNA damage response, and direct cytotoxicity. Lower doses of decitabine (45 mg/m^2^/day, every 8 h, for 3 consecutive days, every 6 weeks) were subsequently found to have an overall response rate of 49% in patients with high-risk MDS in phase II studies^[76,77]^. Subsequent phase III studies confirmed the clinical effectiveness of decitabine in the treatment of patients with MDS, with an overall response rate of 73% with decitabine *vs*. 0% with supportive care in the initial phase III trial^[78]^, and 54% with decitabine in a follow-up phase II trial in elderly patients with intermediate- or high-risk MDS^[79]^. Additional randomized trials have also shown a survival advantage in patients receiving azacitidine, with a median survival of 18 months in the azacitidine arm *vs*. 11 months in the supportive care arm in the Cancer and Leukemia Group B (CALGB) 9221 trial^[80]^, and 24.5 months *vs*. 15 months in patients with intermediate-2- or high-risk MDS in the AZA-001 trial^[32]^.

As indicated earlier in the Introduction, HMAs are also used to treat AML. Azacitidine monotherapy was first evaluated at 150–200 mg/m^2^/day for five days in phase I and II studies in the pediatric patient population with AML and acute lymphoblastic leukemia^[81]^. Six out of the 14 patients with refractory AML achieved remission, but prolonged marrow suppression was a significant adverse event^[81]^. Prolonged myelosuppression was also dose limiting in a study of 154 adult patients with refractory AML treated with 150–700 mg/m^2^/day for 1–7 days^[82]^. After a hiatus of several decades, decitabine and azacitidine were studied again in patients with AML, but at the lower doses that proved effective in MDS. In the AZA-001 trial, analysis of outcomes in a subgroup of patients with 20%−30% bone marrow blasts, who were originally classified as RAEB-t (i.e., refractory anemia with excess blasts in transformation) and subsequently considered as AML by the 2000 World Health Organization classification^[83,84]^, indicated prolonged median overall survival with azacitidine (24.5 months) compared to conventional care regimens such as LDAC, conventional chemotherapy, or best supportive care (16.0 months)^[83]^. An analysis of three CALGB trials with azacitidine 75 mg/m^2^/day for seven days every 28 days by intravenous (CALGB 8421) or subcutaneous routes (CALGB 8921 and CALGB 9221) showed an overall response rates of 36%−48% and median survival of 19.3 months in the azacitidine group compared to 12.9 months for the observation group^[85]^. Retrospective data from European registries showed median overall survival estimates of 9–10 months^[86,87]^. A large phase III study, AZA-AML-001, randomized patients older than 65 years and with > 30% bone marrow blast counts to azacitidine (75 mg/m^2^/day subcutaneously for 7 days every 28 days) or conventional care regimens^[22]^. Although there was no statistically significant difference in survival in the overall study population (10.4 months for azacitidine *vs*. 6.5 months for conventional care), survival was significantly prolonged in the subgroups with poor-risk cytogenetics or AML with myelodysplasia-related changes^[22]^, leading to the European Medicines Agency approval of azacitidine for the treatment of AML in adults above 65 years of age^[22]^. A phase III study (DACO-16) that randomized 485 patients age 65 years or older to decitabine (20 mg/m^2^/day intravenously for 5 days every 28 days) or conventional care regimens (supportive care or LDAC) likewise failed to show a significant improvement in the primary endpoint of overall survival (7.7 months *vs*. 5.0 months, respectively) but showed a higher rate of CR/CRp (CR with incomplete platelet recovery) with decitabine (17.8% *vs*. 7.8%, respectively; *P* = 0.001)^[24]^, leading to approval of single-agent decitabine for the treatment of AML in the older adults in Europe. More recently, the U.S. Food and Drug Administration (FDA) in September 2020 approved once-daily oral azacitidine as maintenance therapy for the continued treatment of AML in adults who achieve their first CR/CRi with induction chemotherapy and are not candidates for further intensive consolidation therapy with HSCT^[71,72]^.

A common theme across the clinical trials of HMAs in AML is a consistent trend towards improved survival despite the low CR rates of < 20%. This led to a number of studies examining the impact of adding various agents to HMAs, ultimately resulting in the HMA/venetoclax regimen described in a subsequent section of this review.

### Mechanisms of resistance to hypomethylating agents

When HMAs are used as single agents in myeloid malignancies, rates of remission are low, and durations of response are often short. Resistance, whether primary or secondary, is a central challenge in the treatment of MDS and AML. Therefore, potential mechanisms of resistance to HMAs have been extensively investigated. In brief, for HMAs to be active, they must accumulate in cells, undergo phosphorylation to the nucleoside triphosphates, avoid metabolic inactivation, and get incorporated into DNA. Resistance has been observed at each of these steps.

Resistance to HMAs can be mediated by membrane proteins that are involved in drug uptake. Human equilibrative nucleoside transporter-1 (hENT1) facilitates transport of nucleosides, including decitabine and azacitidine, in a bidirectional manner across the cell membrane^[88]^. Consistent with a role for hENT1 in HMA cellular uptake, the level of hENT1 mRNA expression is significantly higher in patients who respond to decitabine^[89]^. Interestingly, the decreased activity of azacitidine in cells with low hENT activity can be overcome by the use of an elaic ester of azacitidine, CP-4200, with reduced dependence on membrane transporters^[90]^.

The balance between HMA phosphorylation and enzymatic inactivation also plays a role in response. Cell lines that are resistant to decitabine have not only low expression of hENT1 and hENT2 but also low expression of DCK and high expression of CDA^[36,89]^. Both of these changes are thought to play a role in HMA resistance. Low expression of DCK diminishes the activating phosphorylation of decitabine, and high CDA results in rapid catabolism of decitabine and azacitidine to uridine counterparts that do not contribute to DNMT inhibition^[91,92]^. Consistent with these observations, malignant cell lines engineered to overexpress CDA are decitabine resistant. Azacitidine and decitabine have poor oral availability, likely reflecting high CDA expression in gastrointestinal and liver cells^[91,93,94]^.

In the clinical setting, it has been reported that lower expression of UCK1 is associated with blunted response to azacitidine^[95]^ and that DCK is significantly reduced in the bone marrow and peripheral blood specimens from MDS patients who relapsed after decitabine^[89]^, highlighting the importance of metabolic activation in the action of HMAs. These findings, however, have been far from universal. It has also been reported that expression of DCK or CDA does not distinguish MDS cases that respond to decitabine from those that do not, but the ratio of CDA to DCK was elevated in nonresponding cases^[96]^.

Emerging evidence suggests that metabolism of pyrimidines, including HMAs, reflects an adaptive network of enzymes that detect nucleotide levels and respond in a compensatory manner [Figure 1]^[35]^. For example, DCK protein is diminished in MDS samples with decitabine resistance and upregulated in azacitidine-resistant cells. Conversely, UCK2 protein is upregulated in decitabine-resistant cells and diminished in azacitidine-resistant cells^[35]^. These observations raise the possibility of tailoring therapy based on DCK and UCK levels or diminishing HMA resistance by alternating HMAs^[35,97]^.

After HMAs are metabolically activated, they are incorporated into DNA in place of dCTP. The enzyme carbamoyl-phosphate synthetase (CAD) facilitates the synthesis of dCTP, which competes with aza-dCTP, for incorporation into DNA. Notably, CAD has been found to be upregulated in both cell lines and clinical MDS samples that are resistant to HMAs, although this has not been a universal finding^[35,98,99]^.

A number of additional determinants of response to HMAs have also been identified. Mutations in genes regulating methylation, including TET2, IDH1/2, and DNMT3A, have been associated with improved responses to HMAs in myeloid malignancies^[100–104]^, although this effect has not been universally observed. The variations in conclusions among studies have been attributed to an interaction between different mutations and disease heterogeneity, as suggested by the relationship between lower clonal burden of secondary mutations and higher response rate^[105]^.

As evidence of discordance between DNA methylation status and clinical response accumulated, the effects of HMAs on RNA were also explored^[106]^. About 90% of azacitidine is incorporated into RNA, raising the possibility that alterations in RNA metabolism might contribute to azacitidine action^[107]^. Consistent with this hypothesis, azacitidine response has been linked to NSUN1, an RNA:5-methylcytosine (m5C) methyltransferase that binds BRD4 and RNA-polymerase-II to form chromatin structures that are not sensitive to azacitidine^[108]^. Bone marrow specimens from patients with azacitidine-resistant AML or MDS showed a significant increase in RNA:m5C and NSUN1-/BRD4-associated active chromatin as compared to specimens from patients with sensitive AML or MDS^[108]^. This resistance mechanism is not applicable to decitabine, which contains 5-aza-cytosine bound to 2’-deoxyribose.

## VENETOCLAX

### BCL2 family members and regulation of apoptosis

Apoptosis is a regulated cell death process in eukaryotes that is activated by either extrinsic or intrinsic stimuli. Apoptosis mediates well-conserved decomposition of cellular macrostructures through activation of caspases, cysteine proteinases that cleave next to aspartate residues^[109,110]^. Within the intrinsic pathway of apoptosis, cell death is initiated by mitochondrial outer membrane permeabilization (MOMP) that facilitates the release of cytochrome c, formation of cytosolic apoptosome complexes, and subsequent caspase activation. The MOMP process is regulated by BCL2 family members, which dynamically modulate pro- and anti-apoptotic signaling^[110,111]^. Among the pro-apoptotic signaling proteins are BAK and BAX, which permeabilize the mitochondrial outer membrane upon activation^[110–112]^. These are in turn neutralized when they are bound and sequestered by the anti-apoptotic paralogs BCL2, BCLX_L_, MCL1, BCLW, BCLB, and BFL1^[110–113]^. A third group of BCL2 family members called BH3-only proteins includes BID, BAD, BIK, BIM, PUMA, HRK, NOXA, and BMF, which act through competitive inhibition of the anti-apoptotic BCL2 family members, direct activation of BAK and BAX, or a combination of these two processes^[110–114]^. Upregulation of anti-apoptotic signaling (e.g., through overexpression of BCL2 and its kin) has been implicated in the pathogenesis of cancer, particularly in hematopoietic malignancies, where the upregulation serves as both an essential hallmark of lymphoma- or leukemogenesis and a mechanism of drug resistance^[115,116]^. BCL2 is, therefore, an important therapeutic target for pharmacological inhibition in leukemia and other malignancies^[117–120]^.

### Action of single-agent venetoclax in hematological malignancies

BH3 mimetics are small molecules that have been developed to overcome apoptosis resistance in various neoplasms. These agents were designed to specifically inhibit members of the anti-apoptotic pathway by mimicking the BH3 domain found in BH3-only proteins. Like these pro-apoptotic proteins, BH3-mimetics bind the BH3 binding groove of anti-apoptotic proteins, thereby inhibiting ability of anti-apoptotic family member to bind and neutralize BAX and/or BAK.

The first BH3 mimetic to enter clinical trials was navitoclax (ABT-263), which was designed to specifically mimic BAD binding to BCL2, BCLX_L_, and BCLW^[121]^. Navitoclax showed efficacy in chronic lymphocytic leukemia (CLL) in early human studies^[122]^. However, navitoclax also displayed dose-limiting thrombocytopenia^[123,124]^ that was traced to involvement of BCLX_L_ in platelet survival^[125]^. To avoid this toxicity, venetoclax was engineered to specifically inhibit BCL2 only^[126]^. Venetoclax sensitivity was shown to be BCL2-dependent, with decreased lethality in platelets and nanomolar potency in the BCL2-dependent disease CLL^[122,126–128]^.

As a BH3 mimetic, venetoclax is thought to act primarily by binding to BCL2, causing release of sequestered BAX and BAK, thereby leading to MOMP and apoptosis^[117,119,129]^. More specifically, venetoclax has been shown to form a hydrogen bond at Asp103 in BCL2 and interact with the intercalating indole on a partner BCL2^[126]^. Alternatively, it has also been suggested that venetoclax might also lead to cell death by destabilizing the proton gradient across the mitochondrial inner membrane, leading to acidification of the cytosol and metabolic crisis^[130]^. Whatever the proximate action of venetoclax, the final outcome is MOMP and release of cytochrome c to the cytoplasm, thereby resulting in apoptosome formation as well as decreased ATP production due to diminished mitochondrial cytochrome c^[131,132]^.

Biomarkers that predict response to venetoclax are not fully understood. Venetoclax was initially approved for the treatment of relapsed CLL with chromosome 17p loss^[122]^. High BCL2 protein in this disease^[133]^, which results from deletion of genes encoding two microRNAs that target BCL2 mRNA^[134,135]^, was taken as a sign that CLL might be dependent on BCL2 for survival and, therefore, sensitive to BCL2 inhibition.

The correlation between high expression of BCL2 protein and sensitivity also appears to hold for other neoplasms, but this relationship can be lost when high levels of venetoclax-insensitive BCL2 paralogs such as MCL1 are expressed^[126,136]^. In secondary AML refractory to HMAs, an increase in BCL2 and/or BIM protein detected by immunohistochemistry in myeloblasts in pretreated bone marrow aspirates was strongly associated with venetoclax monotherapy response and overall survival^[137]^. In a phase II, single-arm clinical study of venetoclax monotherapy at a dose of 800 mg daily in patients with relapsed/refractory AML or patients with previously untreated AML who were unfit for conventional chemotherapy, an objective response rate of 19% (6 of 32 patients) was observed, with 6% (2 of 32 patients) achieving CR^[138]^. As described below, high expression of BCL2 relative to other anti-apoptotic BCL2 family members was associated with better outcomes.

### Mechanisms of resistance to venetoclax monotherapy

**Figure 2.**
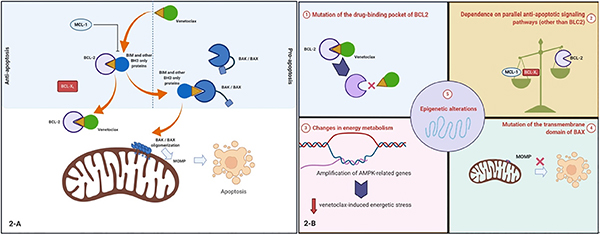
A: proposed mechanisms of resistance to venetoclax in acute myeloid leukemia (AML); B: see text for details: (1) mutations of direct drug-target binding pockets on BCL2. This proposed mechanism has been extrapolated from observations in chronic lymphocytic leukemia; (2) over-expression of MCL1 or BCLX_L_ can cause resistance. For instance, BIM that is released from BCL2 with venetoclax monotherapy can be sequestered by MCL1. This can be reversed by combining venetoclax with cytarabine or daunorubicin, which upregulates the MCL1 binding partner NOXA, or using a selective MCL1 inhibitor; (3) changes in energy metabolism. For instance, upregulation of fatty acid oxidation that may help provide metabolic plasticity to AML cells; (4) mutations of the transmembrane domain of BAX leading to a decrease in BAX-induced MOMP and apoptosis; (5) epigenetic alterations. For example, *HOXA* and *HOXB* genes are highly expressed in highly sensitive samples while resistant samples have little or no expression. Figure was created with BioRender

Mechanisms of resistance in early trials of venetoclax in CLL were well studied^[136,139–142]^. *De novo* resistance was attributable to a lack of BCL2 dependence, either because BCL2 was not activated or the CLL depended upon other paralogs such as MCL1 or BCLX_L_^[143–145]^. Selection of cells that are dependent on an alternative BCL2 family member, such as BCLX_L_ or MCL1, was also a major mechanism of acquired resistance [Figure 2]^[146–148]^.

This mechanism is not limited to CLL. Overexpression of MCL1 and/or BCLX_L_ may play a major role in the pathogenesis of various types of leukemia and mediate venetoclax resistance if BCL2 overexpression is not the primary antiapoptotic driver. In venetoclax-resistant AML cell lines, concurrent inhibition of MCL1 can mitigate venetoclax resistance^[149–151]^. Combining venetoclax with daunorubicin or cytarabine has been shown to reverse some of the BIM sequestration mediated by MCL1, also sensitizing AML to venetoclax^[150]^. In T-cell acute lymphoblastic leukemia cells with both MCL1 and BCL2 overexpression, combination therapy with inhibitors of both MCL1 (S63845) and BCL2 (venetoclax) is highly efficacious^[152]^.

A number of additional mechanisms of venetoclax resistance have been described. One involves the acquisition of mutations within BCL2 itself, such as the Gly101Val mutation, which prevents or reduces venetoclax binding^[136,139–141,153]^. Mutations in the transmembrane domain of BAX have also been shown to prevent mitochondrial anchoring of BAX, thereby preventing BAX from facilitating MOMP and inducing apoptosis^[154]^. In contrast to the BCL2 Gly101Val mutation, which would be expected to affect only drugs that bind BCL2, *BAX* mutations would be expected to confer resistance to a wide variety of agents that activate the intrinsic apoptotic pathway. Another preclinical study using a genome-wide CRISPR/Cas9 screen in AML cell lines identified *BAX*, along with *TP53* and *PMAIP1*, as genes whose inactivation results in venetoclax resistance^[155]^.

A number of resistance mechanisms also involve alterations in mitochondrial metabolism. Amplifications of changes in energy metabolism as a consequence of mitochondrial macrostructure rearrangement also appear to contribute to venetoclax resistance. A genome-wide CRISPR-Cas9 knock-out screen in the AML cell line MOLM-13 identified the mitochondrial chaperonin *CLPB* as a determinant of venetoclax sensitivity^[132]^. Expression of *CLPB* was shown to be significantly higher in AML cells, even greater in cells resistant to venetoclax, which resulted in tighter mitochondrial cristae. Conversely, *CLPB* loss was shown to restore venetoclax sensitivity^[132]^. From a genome-wide CRISPR-Cas9 knock-out screen in a venetoclax-resistant MOLM-13 derivative, the top hits that restored venetoclax sensitivity (*DAP3*, *MRPL54*, *MRPL17*, and *RBFA*) pointed to inhibition of mitochondrial translation as a potential mechanism of sensitizing resistant cells to venetoclax^[156]^. Further studies with doxycycline and tedizolid, which pharmacologically inhibit mitochondrial translation, led to the suggestion that diminished mitochondrial protein synthesis leads to respiratory dysfunction, which activates an integrated stress response that overcomes the resistance^[156]^, possibly by upregulating the pro-apoptotic BCL2 family members NOXA (*PMAIP1*) and PUMA (*BBC3*)^[157]^.

Epigenetic changes also appear to contribute to venetoclax resistance. Mantle cell lymphomas that were resistant to single-agent venetoclax therapy exhibited *TP53*, *SMARCA4*, *CELSR3*, *CCND1*, and *KMT2D* alterations, and allele loss often correlated with super-enhancer remodeling at 18q21^[158]^. Next-generation sequencing or whole exome sequencing of 29 of the 32 cases of AML enrolled in the phase II venetoclax monotherapy study suggested that *SRSF2/ZRSR2* and *IDH1/2* mutations may predict sensitivity to venetoclax therapy in AML^[138,159]^. The effect of *SRSF2* mutation on venetoclax sensitivity may be explained by the alternative splicing of genes involved in apoptotic pathways^[160]^.

In summary, mechanisms of resistance to venetoclax monotherapy are varied, from mutation of the drug binding pocket of BCL2 itself, to amplification of parallel anti-apoptotic signaling pathways, changes in energy metabolism, and epigenetic alterations.

## COMBINATION THERAPY WITH HYPOMETHYLATING AGENTS AND VENETOCLAX

### Mechanism of action

Combination therapy is employed to increase treatment efficacy beyond the action of a single agent, avoid dose-dependent adverse events, and evade potential mechanisms of resistance. With these goals in mind, the combination of venetoclax and HMAs was explored as a potential drug combination in preclinical studies. Treament of clinical AML samples initially *ex vivo* revealed synergy, at low nanomolar concentrations, between azacitidine and venetoclax^[161,162]^. Azacitidine treatment in AML cell lines was also shown to cause several pro-apoptotic changes, including a decrease in MCL1 protein levels^[163,164]^ and an increase in NOXA through activation of the integrated stress response^[157]^. Combination therapy with azacitidine and ABT-737, an inhibitor of BCL2, BCLX_L_, and BCLW, not only displayed synergistic killing of AML cells but also decreased tissue invasion of leukemic cells *in vivo*, thus dampening a potential niche microenvironment-based evasion mechanism of resistance in AML^[164]^. Other preclinical studies have shown that the HMA/BCL2 inhibitor combination decreases oxidative phosphorylation and avoids genomic amplifications that reduce venetoclax monotherapy-induced energetic stress^[165]^. The HMA/venetoclax combination was made even more attractive by the observation that the cytotoxic synergy occurs preferentially in neoplastic cells while sparing normal hematopoietic cells^[166]^.

Based on these preclinical findings, DiNardo *et al*.^[19]^ evaluated the safety and efficacy of the combination of azacitidine or decitabine with venetoclax in previously untreated AML patients 65 years of age or older who were ineligible for conventional induction chemotherapy. The most common grade 3 and 4 adverse events included neutropenic fever, pneumonia, leukopenia, neutropenia, anemia, and thrombocytopenia^[19]^. With a median time on study of ~9 months and a median duration of follow-up of ~15 months, the CR/CRi rate was 67% and the overall leukemia response rate, including patients with partial response and morphologically leukemia free state, was 83%^[19]^. Median overall survival was not reached at the time of the initial report^[19]^. Very similar results were observed in the phase III study of azacitidine and venetoclax in treatment-naïve AML patients ineligible for standard induction therapy^[28]^. Based on the phase II and III HMA/venetoclax results, as well as a phase II study of LDAC that also showed an increased CR/CRi rate and relapse-free survival compared to LDAC alone^[20]^, the combination of venetoclax with HMA or LDAC was approved by the U.S. FDA for the treatment of elderly or medically unfit patients with AML.

### Resistance to combination therapy

The phase II HMA/venetoclax trial^[19]^ and the phase III azacitidine/venetoclax trial^[28]^ both indicate that 1/3 of patients with newly diagnosed AML fail to achieve a CR or CRi with this therapy^[19,28]^ This high rate of treatment failure has prompted investigation into mechanisms of resistance to the combination.

Based on the mechanism of action of HMAs and venetoclax, it is logical that resistance might involve changes in BCL2 family members. Results of Pei *et al.*^[167]^ suggest that resistance to the azacitidine/venetoclax doublet is mediated by the presence of monocytic AML cells, which upregulate MCL1 to evade BCL2 blockade and relieve mitochondrial energetic stress. These monocytes, which lose BCL2 expression and preferentially rely on MCL1 for survival, show outgrowth in relapsed/refractory AML and have a distinct immunopathogenic signature (CD45-bright/SSC^high^/CD117^−^/CD11b^+^/CD68^+^) that might allow early detection and therapeutic decision making^[167]^. Additional analysis showed that AML with monocytic differentiation (previously called M5 AML under the French-American-British classification system) had little or no response to the combination of azacitidine and venetoclax^[167]^.

Another approach to understanding resistance has involved genomic characterization of AMLs that exhibit sensitivity *vs*. resistance. Through genomic analysis of AMLs treated on studies with venetoclax-based combinations, DiNardo *et al*.^[168]^ found that higher response rates and durable remissions were associated with *NPM1* or *IDH2* mutations. AMLs that did not respond to venetoclax-based combination therapy (primary resistance) or initially responded and then relapsed (adaptive or secondary resistance) had new or expanded clones with activating mutations of *FLT3* or *RAS*^[168]^. The variety of mutations occurring in different kinase pathways (e.g., *FLT3*-TKD, *FLT3* N676, *RAS*, or *CBL* among others) and the increase in tumor heterogeneity following treatment indicate that resistance is likely driven by an evolving genetic diversity in the cell pool rather than a single dominant gatekeeper mutation. Genomic changes between diagnosis and relapse involved mutations in a variety of pathways, including kinase signaling such as *FLT3*-ITD, *NRAS*, and *JAK1* mutations; alternative RNA splicing due to mutations in *U2AF1*, *U2AF2*, *SRSF2*, and *ZRSR2*; cancer-related transcription factors, including *IKZF1*, *SETBP1*, *RUNX1*, and *STAT5A*; tumor suppressors such as *TP53* and *WT1*; and epigenetic modifiers such as *BCOR* and *CREBBP*^[169]^. The observed increase in AML cells harboring a *FLT3*-ITD mutation following treatment may indicate a clonal selection in which a small pool of *FLT3*-ITD cells are able to selectively withstand treatment and repopulate the marrow. The addition of inhibitors able to target mutated *FLT3* such as sorafenib or midostaurin to the HMA/venetoclax combination treatment may help to combat this particular form of resistance.

While some of these mechanisms of resistance can potentially be targeted therapeutically (e.g., *FLT3* mutation or even possibly *TP53* mutation), others such as monocytic differentiation are more difficult to directly address pharmacologically. Accordingly, targeting a more general mechanism of resistance that is not usually dependent on specific genetic mutations may be of clinical utility. AML cells take advantage of stromal-dependent pro-survival signals to create a permissive niche within the bone marrow microenvironment via interactions between upregulated cell surface receptors, including very late antigen-4 (VLA-4), CD44, E-selectin ligand-1, and CD98, with adhesion molecules, including vascular cell adhesion molecule-1, fibronectin, hyaluronan, osteopontin, selectins, and integrins. This niche is favorable for the maintenance and progression of chemotherapy-resistant disease^[170,171]^. This mechanism of resistance, although less specific to the topic of this review, may be potentially modified or targeted; treatment with anti-VLA-4 antibodies and cytarabine improves survival in AML mouse models relative to cytarabine alone^[172]^. Additionally, patients with VLA-4-negative AML have a more favorable prognosis^[172]^.

Refined understanding of molecular determinants of sustained response, disease relapse, and secondary resistance to venetoclax-based combination therapy will undoubtedly help to guide future strategies for: (1) monitoring disease status; and (2) devising effective maintenance or salvage therapies. Multiple venetoclax-based combinations are currently being tested with the goal of circumventing drug resistance. These agents added to the HMA/venetoclax doublet include: a combination of cladribine and low-dose cytarabine, the NEDD8-actviating enzyme inhibitor pevonedistat, the anti-CD33 antibody/calicheamicin conjugate gemtuzumab ozogamicin, MDM2 inhibitors, and the anti-programmed death ligand-1 (PDL-1) antibody avelumab^[173]^. How safe and effective any of these strategies are remains to be determined. Despite the relatively high response rates with HMA/venetoclax combinations in patients newly diagnosed with AML, the fact that most responders relapse despite ongoing therapy further underlines the importance of understanding the mechanisms of acquired resistance^[19,28]^. Other outstanding questions include the optimal duration of therapy with the HMA/venetoclax doublet in responders, the effects of treatment interruption or discontinuation on long-term disease control, and the efficacy of the HMA/venetoclax doublet when reintroduced at the time of relapse.

## CONCLUSION

The HMA/venetoclax combination represents are major advance for AML patients who previously fared poorly with conventional induction chemotherapy. As this combination is increasingly used, studies evaluating the causes of loss of response or relapse in clinical samples are needed to elucidate the patterns and mechanisms of resistance. It also remains crucial to explore modified dosing schedules for HMAs as a strategy to avoid acquired HMA resistance based on the results of recent studies with single-agent decitabine and azacitidine.
